# Supplementary material for: Piezoelectric Properties of 0-3 Composite Films Based on Novel Molecular Piezoelectric Material (ATHP)2PbBr4
Source: Materials (Basel). 2022 Sep 14;15(18):6378. doi: 10.3390/ma15186378 (PMC9502484; doi:10.3390/ma15186378)
Supplement: Supplementary file 1 [file materials-15-06378-s001.zip › materials-1899129-supplementary.pdf]

materials MDPI

*Supplementary Materials*

# Piezoelectric Properties of 0-3 Composite Films Based on Novel Molecular Piezoelectric Material $(ATHP)_2PbBr_4$

**1. Preparation apparatus:**

1. Temperature adjustable drying oven: Model No. JK-DOV-9420A, the manufacturer is Shanghai Jingxue Scientific Instruments Co.
2. Electronic balance scale: Model FA1004, the manufacturer is TianmaHengji Company.
3. Intelligent homogenizer: Model CPN-KW-6A type homogenizer, the manufacturer is Zhong Puni Company.
4. ultrasonic vibration cleaner: model JP-010T, the manufacturer is Shenzhen Clean Union Cleaning Equipment Co.
5. drawer type UV ozone machine: model BZS250GF-TC, the manufacturer is (China) Shenzhen Huiwo Technology Co.
6. temperature-controlled digital display magnetic stirrer: model number SP88854105, manufacturer for Thermo Scientific.
7. Intelligent temperature-controlled heating table (hot plate): Model No. BP-2B, the manufacturer is (China) Beijing Heshixingye Technology Co.

**2. Preparation of $(ATHP)_2PbBr_4$ powders:**

According to the amount of $(ATHP)_2PbBr_4$ powder required for the experiment, 0.04 mol of 4-aminotetrahydropyran solution of 4.14 ml and 0.02 mol of lead bromide solid powder of 7.342 g were firstly taken, and then a quantitative amount of 4-aminotetrahydropyran solution and lead bromide solid powder was poured into a 50 ml beaker, and a total of 8 ml of hydrogen bromide solution was taken with a pipette gun. After that, the residual mixture on the beaker wall was cleaned off with a glass rod and put into a suitably large magnetic stirrer, and then the mouth of the beaker was tied tightly with a breathable cloth and put on a temperature-controlled digital magnetic stirrer, and the temperature was adjusted to 90 °C with a suitable After about 24 hours, the solvent evaporated and the remaining yellowish solid powder was the $(ATHP)_2PbBr_4$ powder required for the experiment. Since this material is in powder form, the precipitated powder needs to be transferred to a vial, sealed with a sealant, and stored in a dry environment at room temperature.

**3. Cleaning of ITO conductive substrates:**

The cleaning steps of ITO glass are as follows:

(1) The marked ITO glass is put into a Petri dish, and the detergent diluted with deionized water is added. The ITO glass surface is brushed one by one with a small brush and rinsed with flowing deionized water. This step can initially remove the dust and organic stains from the ITO conductive surface.

(2) The rinsed glass is placed into separate small recesses of the glass holder, and the glass holder is placed into a suitable-sized beaker with the diluted detergent, which is not covered by the ITO glass sheet. The beaker with the glass pieces was placed into the recess of the ultrasonic cleaner, and the recess was poured with an appropriate amount of water in advance to act as the ultrasonic medium. After the ultrasonic time is set to 30 min, take out the holder, pour off the liquid in the beaker, and rinse it repeatedly with flowing deionized water to remove the residual detergent.

(3) Acetone, isopropyl alcohol, and deionized water were configured into a glass cleaning solution at a volume ratio of 1:1:1, and poured into the beaker with the glass piece, again without covering the glass, for the 3rd cleaning. This step can remove the stains on the ITO glass that can be dissolved in alcohol and acetone.

(4) After finishing the 3rd cleaning, pour off the mixed liquid in the beaker and rinse it once with anhydrous ethanol. Then pour in fresh anhydrous ethanol and store it without the glass.

**4. Supporting Figures:**

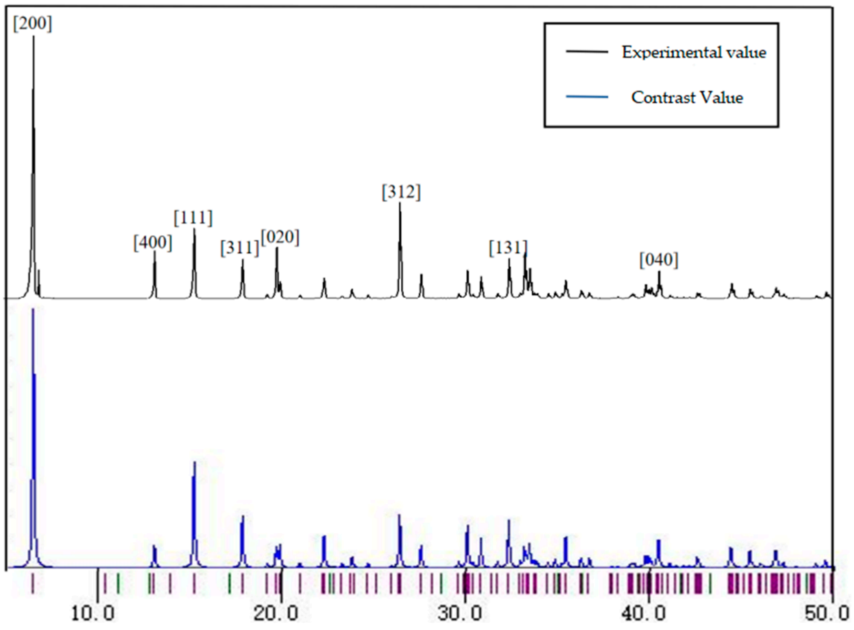


**Figure S1.** XRD pattern of $(ATHP)_2PbBr_4$.

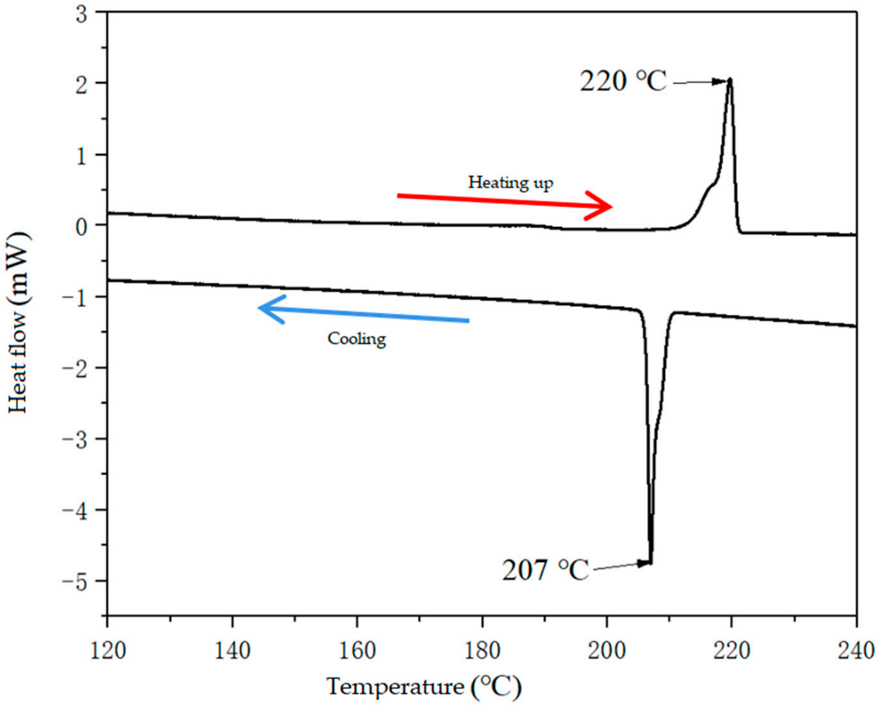


**Figure S2.** DSC diagram of $(ATHP)_2PbBr_4$ powder.

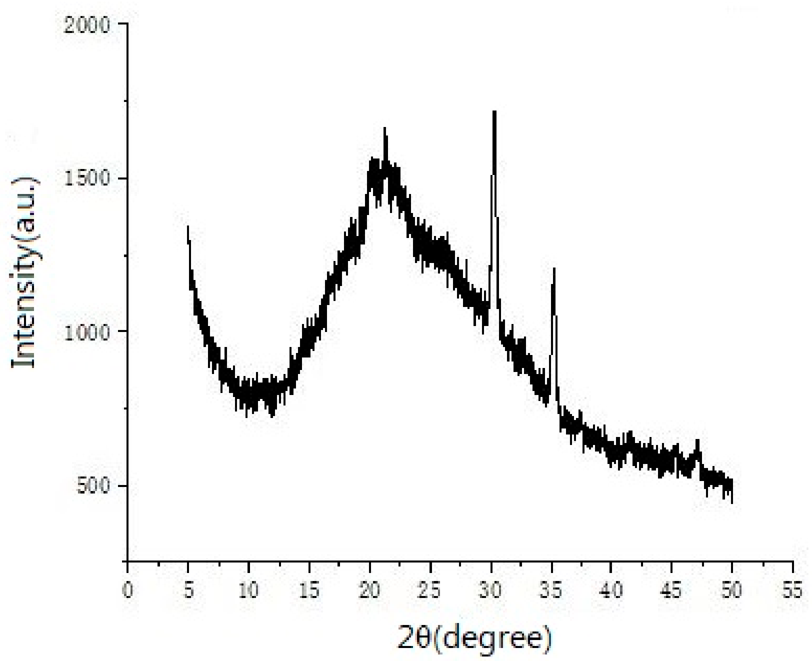


**Figure S3.** XRD pattern of PVDF film at an annealing temperature of 90 °C at 500-1000 r.p.m. speed.
